# Detection of Hindwing Landmarks Using Transfer Learning and High-Resolution Networks

**DOI:** 10.3390/biology12071006

**Published:** 2023-07-14

**Authors:** Yi Yang, Xiaokun Liu, Wenjie Li, Congqiao Li, Ge Ma, Guangqin Yang, Jing Ren, Siqin Ge

**Affiliations:** 1 Key Laboratory of Zoological Systematics and Evolution, Institute of Zoology, Chinese Academy of Sciences, Beijing 100101, China; 2 University of Chinese Academy of Sciences, Beijing 100049, China; 3 State Key Laboratory of Plant Genomics, Institute of Genetics and Developmental Biology, Chinese Academy of Sciences, Beijing 100101, China

**Keywords:** neural network, leaf beetle, hindwing, landmark, morphology

## Abstract

**Simple Summary:**

The landmark annotation of hindwing venation is one of the most important methods in the hindwing morphological, functional, and evolutionary analysis of beetles, and the number of the landmark samples greatly affects the effectiveness of these analysis. However, large-scale manual landmark annotation is a time-consuming task that hinders the progress of wing morphology research. Some machine learning techniques have been applied to beetle image recognition, but the lack of data for beetle hindwing landmarks limits the use of machine learning for beetle hindwing landmark detection. In this study, we propose a new approach to solve the problem of insufficient training samples for beetle hindwing landmark detection, by fine-tuning a new deep high-resolution convolutional neural network pretrained on a natural image database to transfer it to the domain of beetle hindwings. The results of experiments shows the effectiveness of this new approach as it demonstrated small error in the detection of leaf beetle hindwing landmarks and required a very low number of samples.

**Abstract:**

Hindwing venation is one of the most important morphological features for the functional and evolutionary analysis of beetles, as it is one of the key features used for the analysis of beetle flight performance and the design of beetle-like flapping wing micro aerial vehicles. However, manual landmark annotation for hindwing morphological analysis is a time-consuming process hindering the development of wing morphology research. In this paper, we present a novel approach for the detection of landmarks on the hindwings of leaf beetles (Coleoptera, Chrysomelidae) using a limited number of samples. The proposed method entails the transfer of a pre-existing model, trained on a large natural image dataset, to the specific domain of leaf beetle hindwings. This is achieved by using a deep high-resolution network as the backbone. The low-stage network parameters are frozen, while the high-stage parameters are re-trained to construct a leaf beetle hindwing landmark detection model. A leaf beetle hindwing landmark dataset was constructed, and the network was trained on varying numbers of randomly selected hindwing samples. The results demonstrate that the average detection normalized mean error for specific landmarks of leaf beetle hindwings (100 samples) remains below 0.02 and only reached 0.045 when using a mere three samples for training. Comparative analyses reveal that the proposed approach out-performs a prevalently used method (i.e., a deep residual network). This study showcases the practicability of employing natural images—specifically, those in ImageNet—for the purpose of pre-training leaf beetle hindwing landmark detection models in particular, providing a promising approach for insect wing venation digitization.

## 1. Introduction

As one of the most vital functional organs of insects, with implications for insect flight, wings are considered a key factor in the evolutionary success of insects [1,2]. The wing morphology, such as the wing length, wing shape, tip shape, and radial sector shape, reflects the functional adaptation [3,4,5] and evolutionary history of insects [6,7]. Beetles are the most diverse group of insects, and both their forewing specialization and hindwing folding [8] are regarded as meaningful key morphological indicators that are indispensable in the analysis of wing morphology evolutionary patterns [9].

Wing landmark annotation based morphological methods have been widely used in studies on beetles, in terms of aspects such as phylogeny [10], evolution [11,12], ecology [13], biogeography [14], and flight deformation [15]. The amount of landmark data on the wing largely determines the effectiveness of the subsequent morphological analysis [16]. However, with the use of software for the manual annotation of landmarks (e.g., tpsDig [17]), obtaining digitized morphological landmark datasets from a large volume of insect wing pictures is highly time-consuming. Hence, it is imperative to incorporate machine learning technology in order to address the problem of detecting landmarks in a large amount of beetle hindwing images.

Machine learning has already been used in insect morphology research, mainly for species identification [18,19]. The deep residual network (ResNet), as a convolutional neural network (CNN) and also one of the most prevalent machine learning methods at present, can be used to identify species of beetles [20]. To date, few machine learning methods have been used for the landmark detection of insect wings [21,22], especially beetle hindwings. In addition, conventional network training methods currently cannot obtain effective models on small training datasets, given the lack of publicly available large-scale beetle hindwing landmark datasets.

To solve the problems outlined above, and to improve the efficiency of obtaining landmarks and reliability of the subsequent morphological analysis, we propose the combination of the deep high-resolution network (HRNet) and transfer learning to detect leaf beetle hindwing venation landmarks. HRNet, as a derived type of CNN with high resolution, performs well in terms of landmark detection [23], but is dependent on a training set with a large number of leaf beetle hindwing samples. Transfer learning, which has previously been applied for beetle classification [20,24], can compensate for the inadequacy of hindwing data pertaining to leaf beetles through the migration of pre-trained models based on a readily available dataset [25,26]. ImageNet, which is presently the largest database for natural image recognition worldwide, comprises over 14,000,000 images and 20,000 categories (accessed on 11 April 2023 via http://www.image-net.org) [27], and is commonly used for pre-training in transfer learning [28]. Through the process of fine-tuning, the HRNet model parameters pre-trained with ImageNet on leaf beetle hindwing landmark datasets, we tested the detection performance for 36 landmarks on the leaf beetle hindwing venation with an extremely restricted number of training samples. The model exhibited promising usability for hindwing landmark detection. The main innovations of this study are as follows: (1) We created a hindwing landmark dataset for the family Chrysomelidae (HWLFC); (2) We combine HRNet with transfer learning to detect leaf beetle hindwing landmarks automatically. This approach provides a novel perspective for better understanding of the evolution of beetle hindwings.

## 2. Materials and Methods

### 2.1. Hardware and Software Environment

For the present investigation, we utilized Python 3.8.10 (developed by the Python Software Foundation; Beaverton, OR, USA) and PyTorch 1.12 (developed by Facebook, Inc.; Menlo Park, CA, USA) as foundational programming languages for both training and testing procedures. We employed the Adam algorithm [29] to optimize the network parameters, along with the learning schedule described in Ref. [30]. The base learning rate is set to 0.0001 [31], and is dropped to 0.00001 and 1 × 10${}^{-6}$ at the 40th and 55th epochs, respectively. The batch size was set to 8 and the training process was terminated within 80 epochs. We ran it on a graphics workstation with the Ubuntu 20.04.4 LTS OS, an AMD Ryzen Threadripper 2990WX 32-Core Processor, 128 GB RAM, and an NVIDIA GeForce RTX 2080Ti GPU. The versions of CUDA and cuDNN (both developed by the NVIDIA Corporation; Santa Clara, CA, USA) were 11.6 and 8.3.2, respectively.

### 2.2. Image and Dataset Preparation

**Figure 1:**
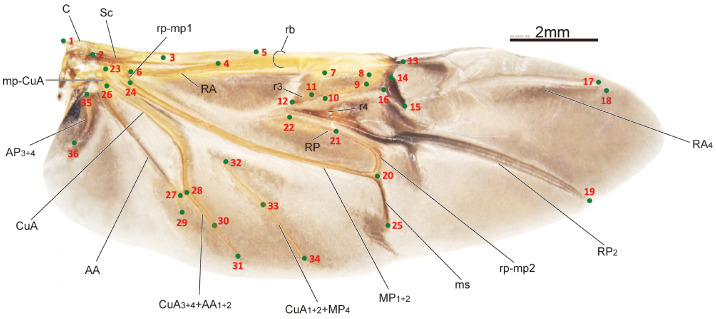
The distribution of the hindwing landmarks and the names of the hindwing veins for the leaf beetle (*Potaninia assamensis*).

The sample processing and imaging of the leaf beetle hindwings used in this study have been described in detail in one of our previous papers [32]. Briefly, the hindwings of the specimens were obtained through examination and dissection using a LEICA MZ 12.5 dissecting microscope. Subsequently, images of the hindwings were captured using a Nikon D500s camera connected to a Zeiss Stereo Discovery V12 stereoscope. The HWLFC dataset contains images of 256 leaf beetle hindwings, representing 16 subfamilies and 231 genera. The principle of landmark selection is based on key points in the hindwing vein distribution, such as intersections, bases, and ends or beginnings of hindwing veins. The HWLFC dataset includes the same 36 landmarks (Figure 1, Table 1), and the annotation data are formatted according to the COCO dataset [33].

The annotated dataset consists of two parts: Image information and annotation information of landmarks. The image information includes the index (denoted as “id”), file path (denoted as “file_name”), width (denoted as “width”), and height (denoted as “height”) of each image. The annotation information of landmarks contains several fields. The landmark coordinate array (denoted as “Keypoints”) has a length of 3k, where *k* is the number of landmarks on a leaf beetle hindwing. Each group of three components corresponds to a single landmark, with the first two components representing the *x* and *y* coordinates, respectively, and the third component representing a visibility flag bit (*v*) which is not used in HWLFC as all landmarks are visible. The landmark number (denoted as “num_keypoints”) indicates the total number of landmarks for a leaf beetle hindwing. The bounding box of the hindwing (denoted as “Bbox”) denotes the location of the hindwing in the image, with the first two components representing the upper-left coordinates of the bounding box and the last two components representing the width and height of the bounding box [33].

The images are in TIFF format and have a size of 4288 × 2848 pixels. The coordinates of the landmarks in the measurements take the lower left corner of the image as the origin, with the image height subtracted from the y coordinate of the feature point to obtain the coordinates of the annotation data, taking the top left corner as the origin. The value “num_keypoints” is equal to 36, and “Keypoints” is an array of length 108. “Bbox” represents the coordinates of the top left corner and the width and height of the bounding box in the image, with the distance from landmark 1 to landmark 18 used as the normalized length.

### 2.3. Network Architecture

**Figure 2:**
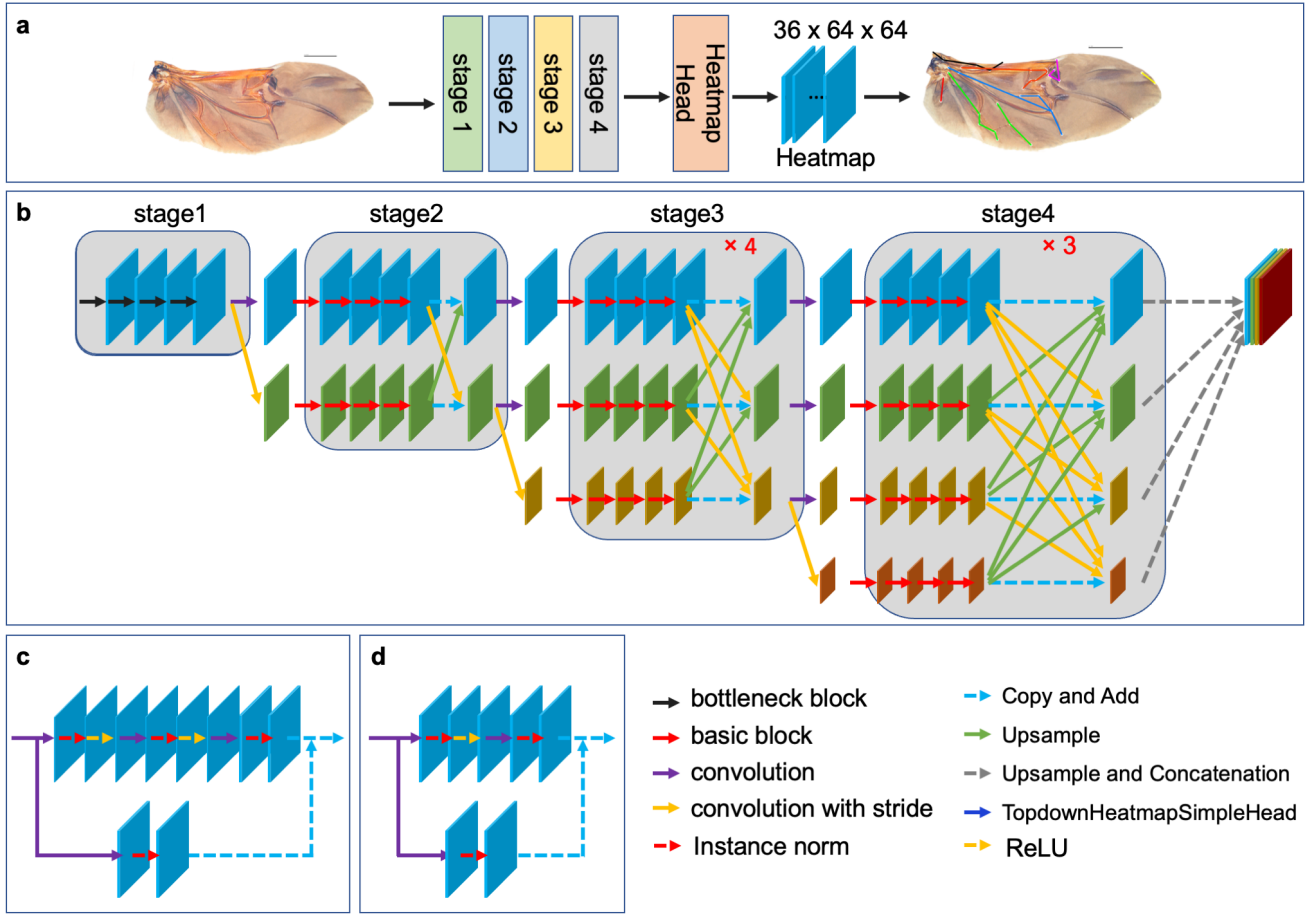
The architecture of HRNet: (**a**) Leaf beetle hindwing landmark detection model based on transfer learning and HRNet; (**b**) the overall structure of HRNet, which consists of four stages. Each stage adds a low-resolution feature channel, the input resolution channels are processed in parallel, the resolution features are combined at the end of each stage, and four feature maps with different resolutions are the output; (**c**) the basic block; and (**d**) the bottleneck block.

In this study, HRNet is used to learn a rich representation of wing morphology and improve the accuracy of hindwing landmark detection. The architecture of HRNet, as shown in Figure 2b, comprises four stages. Stage 1 consists of a high-resolution convolution branch, while subsequent stages add low-resolution convolution branches at the corresponding levels. Additionally, the different resolution convolution branches are parallel to each other, in order to achieve multi-resolution convolution. Stage 1 is composed of four consecutive bottleneck modules (Figure 2d), while Stages 2, 3, and 4 are composed of consecutive basic modules (Figure 2c). A fusion module is included after every four basic modules, in order to achieve multiresolution feature fusion. Both the bottleneck and basic modules are residual units, with residual connections consisting of a convolution with a kernel size of 1 × 1. The forward connection of the bottleneck module contains three convolutional layers with configurations (1 × 1, 1, 0), (3 × 3, 1, 1), and (1 × 1, 1, 0). The number of channels is first reduced and then enlarged. The forward connection of the basic module contains two convolution layers with configuration (3 × 3, 1, 1), and the number of channels remains constant. To facilitate information exchange between multi-resolution representations, Stages 2, 3, and 4 re-use the fusion module to obtain one, four, and three feature fusions, respectively. HRNet is followed by a representation head that concatenates the three low-resolution features into a high-resolution representation through up-sampling. The output of the representation head is then transferred to the top-down heatmap head to generate the predicted heatmap of the landmarks. The dimensions of HeatmapHead are (B, C, H, W), where B is the number of samples in the batch, C is the number of channels (which corresponds to the number of landmarks), and H and W are the height and width of the heatmap, respectively. For the hindwing venation of leaf beetles, C is equal to 36, while H and W are both equal to 64.

For this study, we applied the HRNet-w18 model pre-trained on ImageNet [27,34] and developed a model for detecting landmarks on leaf beetle hindwings through parameter transfer. Specifically, we preserved the network structure and parameters of certain stages, while re-training the network structure of the remaining stages to optimize the parameters using a limited leaf beetle hindwing landmark dataset [35,36]. We implemented three distinct strategies to fine-tune the parameters of the HRNet model, as illustrated in Table 2.

In the training phase, the pretrained weights obtained from the natural image dataset were loaded into the model. The model was then trained on the leaf beetle hindwing landmark dataset. In particular, the pre-training parameters of HRNet obtained from ImageNet were loaded into the backbone network, while the parameters of the heatmap head network were initialized with random values. The hindwing images were loaded into HRNet, generating four feature maps with high to low resolution. The representation head fused these four features of different resolutions into a high-resolution representation through up-sampling and convolution, and the fused representation was input into the heatmap head to obtain the predicted heatmap of landmarks. Mean Squared Error (MSE) loss was then computed using the predicted heatmap and the ground truth heatmap.

## 3. Results

The test set was created by randomly selecting 80 samples from HWLFC, each of which included an image of a leaf beetle hindwing and its corresponding landmark annotation. To ensure the repeatability of the experiment and evaluate the performance of the model on training sets of varying size, the remaining samples were used to construct 10 groups of training sets, each containing a specific number (i.e., 1, 3, 5, 10, 50, 100) of randomly selected samples. To evaluate the performance of the model, we applied the Normalized Mean Error (NME) metric [37], which is a commonly used evaluation metric in landmark detection tasks [38]. The NME is defined as follows:

(1) $$\mathrm{NME}\left(P,\hat{P}\right)=\frac{1}{N_{P}}\sum_{i=1}^{N_{P}}\frac{∥p_{i}-{\hat{p}}_{i}∥}{d},$$

where *P* and $\hat{P}$ represent the predicted and true coordinates of the landmarks, respectively; $N_{P}$ is the number of landmarks, which is 36 in the hindwing of a leaf beetle; and *d* is the reference distance used to normalize the absolute error. The NME metric measures the average Euclidean distance between the predicted and true landmarks, normalized by the reference distance. We used the distance between the proximal anterior point of the humeral plate (Landmark 1) and the distal point of Radius Anterior1 (Landmark 18) as the reference distance for HWLFC. This metric provides a measure of the accuracy of the model’s predictions and allows for quantitative comparison of the performance of different models.

### 3.1. Performance of Transfer Strategies

**Figure 3:**
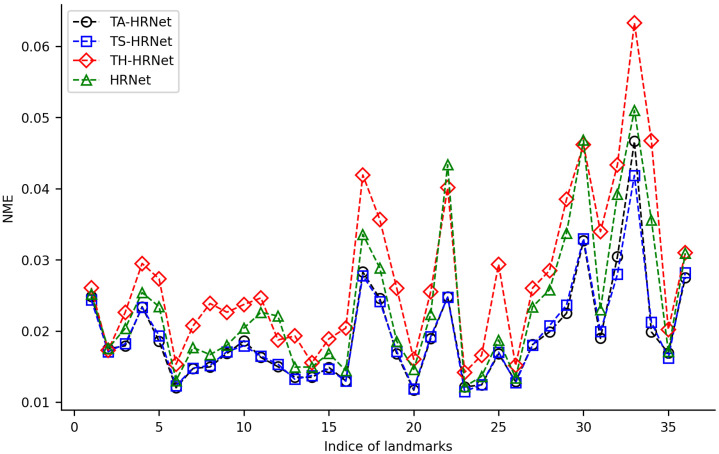
NME changes for 36 landmarks on leaf beetle hindwings using 4 training methods.

**Figure 4:**
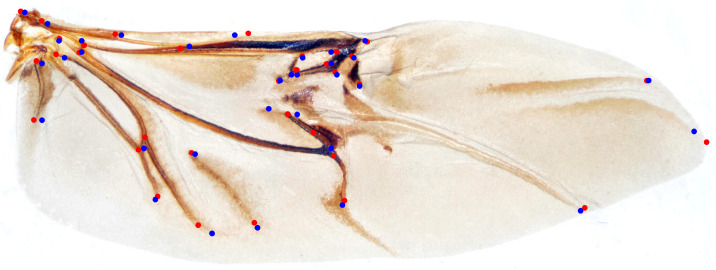
The manually annotated (red) and predicted (blue) landmarks on a leaf beetle hindwing (*Phratora bicolor*).

We randomly selected 100 samples from HWLFC as the training set, then compared the performance differences between the initial HRNet model and three HRNet models with different transfer strategies (TH-HRNet, TS-HRNet, and TA-HRNet in Table 2) using the unique test set (Figure 3 and Table S1 in the Supplementary Materials). The manually annotated (red) and TS-HRNet-predicted (blue) landmarks on a leaf beetle hindwing (*Phratora bicolor*) are shown in Figure 4. Only the results with the optimal strategy of TS-HRNet is detailed in Table S1, in which Stage 1 is retained with parameters as a general feature layer, while Stage 2, Stage 3, and Stage 4 are re-trained with parameters as specific feature layers. The test results for the other strategies are shown in Table S2.

Our experiments show that applying transfer strategies can effectively improve the performance of CNNs, and the average NME values for TS-HRNet and TA-HRNet were better than those of HRNet (Table S1). Among the three transfer strategies, TS-HRNet performed the best, in terms of the NME for each hindwing landmark (0.0115–0.0419; average 0.0193), while TA-HRNet presented evident fluctuations depending on the hindwing landmark, and TH-HRNet had relatively high NME (see Figure 3). In summary, TS-HRNet obtained the best NME and stability in the landmark detection tasks covering different landmarks on the hindwings of leaf beetle. Therefore, in subsequent experiments, we utilized TS-HRNet to test the parameter fine-tuning strategy.

### 3.2. TS-HRNet Performance on Training Datasets of Varied Sizes

**Figure 5:**
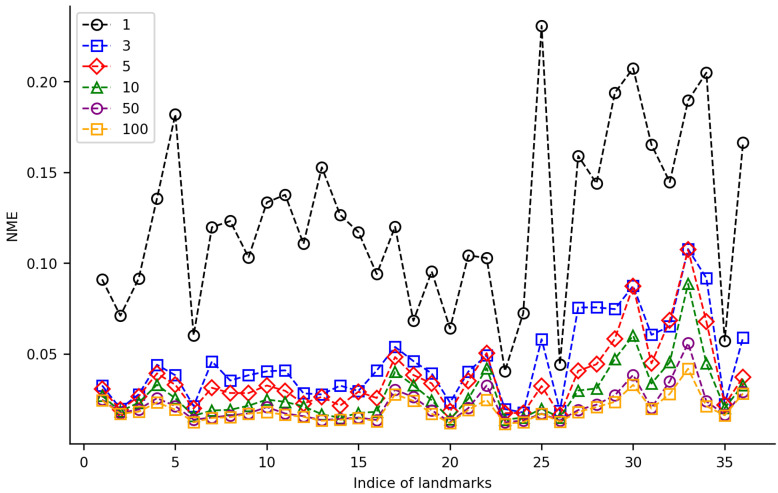
NME values for 36 landmarks on leaf beetle hindwings using TS-HRNet on 6 datasets of varying size.

For the TS-HRNet model, we constructed different training datasets with 1, 3, 5, 10, 50, and 100 samples from HWLFC, then tested the impact of the training dataset size on the landmark detection performance (Figure 5, Table S3). When the training dataset contained 50 or more samples, the average NME of TS-HRNet was less than 0.022. Almost all landmarks were detected accurately in the test images, with only a slight fluctuation in NME (0.0213 ± 0.0091). When the size of the training dataset was reduced to three samples, the NME of most landmarks remained at a low level (<0.046). Nine specific landmarks—namely, 25, 27, 28, 29, 30, 31, 32, 33, and 36—led to an evident reduction in detection efficacy, although the normalized mean error (NME) remained below 0.110. When there was only one sample in the training dataset, the NME of all landmarks increased significantly (average NME was 0.123). As the size of the training dataset increased, there was a notable decrease in the NME of certain landmarks, such as the posterior of medial spur (Landmark 25), whose NME decreased from 0.23 to 0.02 as the size of the training dataset increased from 1 to 10.

### 3.3. Performance Comparison of TS-HRNet with TS-ResNet

In order to verify the performance advantage of TS-HRNet in detecting multiple landmarks on leaf beetle hindwings under small-sample conditions, we conducted an ablation experiment on TS-HRNet with the baseline deep learning method as ResNet. ResNet has been widely used in deep learning research and is well-suited for facilitating transfer learning [31]. Utilizing the ResNet model pre-trained on ImageNet, we obtained the corresponding TS-ResNet by using the identical transfer strategy, parameter configuration, and training and testing datasets as with TS-HRNet, and then recorded the testing results.

**Figure 6:**
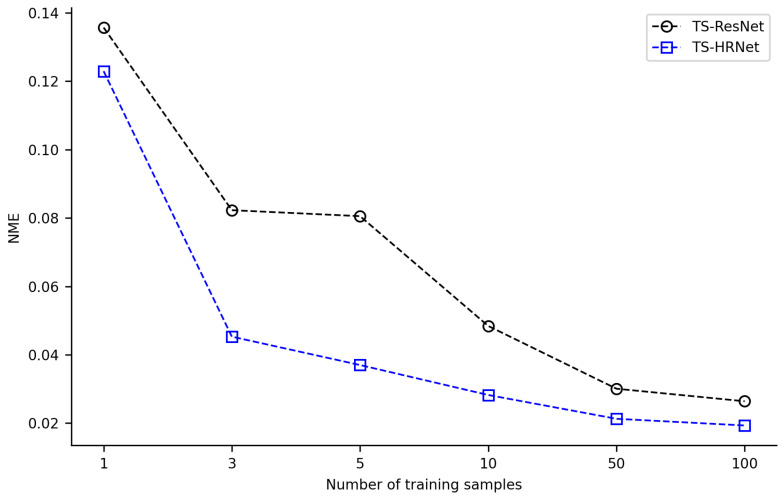
Average NME of TS-HRNet and TS-ResNet under different numbers of training samples.

The combination of transfer learning with TS-ResNet and TS-HRNet was effective in scenarios with few training samples (1 or 3). Acceptable detection NME (<0.03) could be achieved with only a few training samples, where TS-ResNet required about 50 samples, while TS-HRNet required only 10 samples (Figure 6, Supplementary Tables S3 and S4). By expanding the training dataset to over 100 images, the models detected landmarks with very low NME (TS-ResNet: 0.0264, TS-HRNet: 0.0193). Across all scenarios with varying numbers of training samples, TS-HRNet outperformed TS-ResNet in terms of NME.

## 4. Discussion

### 4.1. Entomological Significance of Wing Landmark Detection

The landmark detection of leaf beetle hindwings is important for better understanding the evolution of beetles. Beetle hindwing venation is one of the most important features, in terms of evolution and diversity [15,39], and wing landmarks reflect changes in wing structure and the morphological evolution of beetles and other pterygote insects [40]. For example, the evolution of the forewing shape and size is independent of hindwing shape and size in *Papilio* butterflies, which is reflected by wing landmark data [16]. The variation in wing venation reflects the diversity of beetles [40,41]; for instance, differences in the number and distribution of wing veins are closely related to flight, and feeding habits [42,43]. In addition, wing venation landmarks can be used to analyze the geographical distribution and evolutionary history of insects [7,9]. Differences in wing venation between populations or species reflect their adaptation to various environments. By comparing the wing venation landmarks of different populations or species, their evolutionary relationships and habitat preferences can be analyzed [12].

The distribution of beetle hindwing venation is a key feature for analyzing beetle flight performance, which is important for designing beetle-inspired micro aerial vehicles [44,45]. Hindwing landmarks can provide important information about the structural and functional features of beetle hindwings related to flight performance [12]; for example, the number and position of veins can affect the stiffness and elasticity of the hindwing, which consequently affects the lift and maneuverability of beetles [46,47]. Furthermore, the shape and size of hindwing veins can affect the aerodynamic performance parameters of the hindwings [48], such as drag and lift coefficients [49]. Therefore, by analyzing the hindwing landmarks of different beetle species, we can gain insights into the relationships between wing structure and flight performance, and may identify the key parameters for optimizing the flight performance of beetle-like flapping wing micro aerial vehicle [45,50].

The study of landmarks, especially the joints of wing veins, is the premise of folding mechanisms study and deployable wing design. By automatically detecting the landmarks of hindwing veins with the proposed approach, designing linkage mechanisms by simulating insect veins becomes a feasible solution. A rigid and flexible coupled bionic deployable wing may use linkage mechanisms as artificial veins according to the detected locations of joints to achieve flapping wing folding/unfolding and vein joints rotating [44]. Coordinates of landmarks can be obtained quickly with the proposed approach, and discriminant space of landmark-based wing venation geometric morphometrics can be used to distinguish morphologically similar species to explore intraspecific variation among populations and determine sexual dimorphism in various insects [3]. It can also be used to identify damaged specimens faster than traditional morphological methods and is less expensive than molecular methods [4].

### 4.2. Improving Landmark Detection Models with Transfer Learning

In the computer vision field, deep learning typically requires a large amount of data for training in order to extract useful features from images. When the training dataset is too small, the model may fail to learn landmark features sufficiently, resulting in poor generalization to new datasets and a decrease in performance [51]. Similarly, the performance of a landmark detection model is tightly linked to the size of the dataset; however, for certain tasks such as leaf beetle hindwing landmark detection, the dataset may be very limited, making it difficult to train an accurate model.

Transfer learning is a method that is commonly used to address this problem [20], which involves using a pre-trained model as a starting point and fine-tuning it on a small target task dataset [52]. By enabling the model to maintain its previous knowledge base while learning new feature space, transfer learning provides an effective solution to address dataset scarcity [53]. Ideally, leaf beetle landmark data would be the best pre-training dataset in the present context, however, there are currently no publicly available datasets for leaf beetle hindwing landmarks. ImageNet, which has been widely used in the computer vision field as a publicly available large-scale image data set, has been shown to be effective in pre-training deep learning models for different tasks [54]. The combination of the ImageNet dataset and transfer learning has already shown great potential for beetle species recognition [20,24] and can therefore be used as a pre-training dataset for beetle hindwing landmark detection.

In this study, three different transfer learning models were used to improve the landmark detection performance of leaf beetle hindwings. The results showed that two of the models achieved an average detection error less than 0.02 on the 36 leaf beetle hindwing landmarks (Figure 3), indicating a significant improvement in detection performance. This suggests that transfer learning is particularly useful with a limited dataset, as it allows the model to leverage pre-trained features from other tasks to improve its performance. Through transfer learning the low-level features trained on natural images are also effective for leaf beetle hindwing images. In contrast, models without transfer learning may not be able to adjust all model parameters on limited data and thus may not leverage pre-trained models from other tasks for low-level feature re-usage, leading to sub-optimal weights and poor performance. Therefore, the combination of transfer learning and the HRNet model for leaf beetle hindwing landmark detection presents significant advantages over more traditional approaches. In summary, our results demonstrate that transfer learning can greatly improve the detection performance of CNNs with a small amount of training data for hindwing landmarks, both across domains (i.e., from natural images to leaf beetle hindwing images) and tasks (i.e., from classification to landmark detection). This technology may even be applied to various other insect groups.

There are potential challenges related to domain adaptation and dataset bias in using transfer learning for landmark detection. The morphological features of insect wing veins may vary due to different species, ages, genders, etc., which requires addressing domain adaptation. Moreover, the pre-trained model used for transfer learning may have been trained on a dataset that is biased towards certain features or characteristics, which may not be relevant to the target dataset. This can lead to a decrease in performance and accuracy of the model. Therefore, while transfer learning is a powerful tool for addressing data scarcity in landmark detection, it is important to carefully consider the limitations and challenges associated with this approach and to choose the appropriate method based on the specific requirements of the task at hand. Future research in this area could focus on developing more robust and adaptive transfer learning methods that can effectively address these challenges and improve the performance of landmark detection models.

### 4.3. Performance Analysis of TS-HRNet

In classification, CNNs typically extract high-level features that capture the overall shape, texture, and color of objects or images, which are useful for discriminating between different image categories. Meanwhile, in landmark detection, low-level features that capture the edges, corners, and other location-sensitive features of images are extracted [55], which are meaningful for identifying specific landmarks in the image. Freezing all stages may limit the new-task learning ability of the HRNet model pre-trained on the ImageNet classification task [54], especially for landmark detection requiring significantly different features from the classification task. By fine-tuning some of the stages, the model has a greater capacity to learn new features relevant to the landmark detection task. Therefore, the performance of TH-HRNet was slightly worse than that of HRNet, as it is inconsistent with the classification results of cross-domain transfer learning [31], but TS-HRNet and TA-HRNet performed better than HRNet (Figure 3).

In our experiments, HRNet in combination with transfer learning (TS-HRNet) demonstrated competitive performance in the leaf beetle hindwing landmark detection, even when using a limited training dataset. The average NME remained below 0.045 when the number of training samples was reduced to only 3, and below 0.020 when the sample size was increased to 100 (Figure 5). For one sample, TS-HRNet presented poor detection performance and large fluctuations, although this is an extreme case. Under various training samples of different sizes, the average NME of TS-HRNet in the landmark detection task was significantly lower than that of TS-ResNet, which employed the same transfer learning strategy (Figure 6).

The performance of TS-HRNet is heavily dependent on the amount and quality of the training data. With a relatively small number of samples, the model may not have enough data to learn the complex features of hindwing landmarks, resulting in poor detection performance. This was particularly evident in the case of Landmarks 25, 27–34, and 36 located in the cubitus-anal region (Figure 5, Table 1 and Table S3), which may be too variable to learn with limited data as most of the morphological differences among different sub-families and genera occur in this region of the hindwing [32]. Furthermore, in the membranous zone the veins in the distal region of the leaf beetle hindwing degenerate into traces, rather than tubular structures. This membranization resulted in TS-HRNet achieving low detection accuracy on a few end point landmarks (i.e., Landmarks 17, 18, and 31) and marginal point landmarks (i.e., Landmarks 4 and 5) on the veins that were not obvious (Figure 5, Table 1 and Table S3).

On the other hand, TS-HRNet performed well in detecting landmarks on both radial (R) and medial (M) veins (i.e., Landmarks 2, 3, 6–16, 20, 23, 24, and 26; Figure 5, Table 1 and Table S3). As the hindwing morphology of leaf beetles has not undergone substantial changes throughout the evolution of leaf beetles, the R and M veins—which are crucial for flight as they stabilize the radial and apical wing fields—have remained essentially constant throughout their evolution [32].

Although TS-HRNet showed a reduction in the detection efficacy on a few landmarks in specific regions when the size of the training dataset was reduced to three samples or less, most landmarks still remained at a low NME level. When the training dataset contained 50 or more samples, the average NME of TS-HRNet was consistently low, with almost all landmarks being accurately detected in the test images. Therefore, the accuracy was not significantly affected by the differences in detection performance across different regions as the training dataset increases, and the proposed method is robust and effective for detecting landmarks on leaf beetle hindwings.

### 4.4. Implications for Future Studies

In this study, we detailed the construction of a dataset for the hindwing landmarks of leaf beetles and the combination of HRNet and transfer learning for the detection of these landmarks. Our method accurately predicted the hindwing landmarks (Figure 3), providing valuable data for morphological analysis. The method showed promising results on a sample of limited size consisting of leaf beetle hindwing landmark data (Figure 5). Together with the construction of the hindwing landmark dataset, the proposed approach allows a large amount of beetle hindwing landmark data to be quickly obtained using only a small number of manually annotated samples. The construction of the hindwing landmark dataset and the landmark detection method can be extended to the whole order of Coleoptera and even other insects, providing a more efficient way to study their morphological evolution and diversity.

However, the detection errors for the landmarks in the cubitus–anal area, as well as in the membranous distal area of the leaf beetle hindwings, were relatively large (Figure 3). Further research is required to analyze and understand the structural features that are important for automatic detection of these landmarks, in order to further improve the detection performance on these landmarks. Methods that can be used for this purpose involve the visualization of CNNs, such as random input sampling explanation (RISE) [56], which can visualize the key areas of the method used in this study for wing landmark detection, thus allowing for a better understanding of the important structural features required to detect these landmarks.

Based on the coordinates of landmarks on the leaf beetle and even the beetle hindwings predicted by HRNet in combination with transfer learning, further analyses and studies can be carried out. Procrustes alignment provides the means to minimize the non-shape differences between different samples and obtain Procrustes coordinates for morphological analysis. Furthermore, principal component analysis (PCA) can be applied to analyze the main trends of beetle hindwing evolution [57,58], and the ancestral morphology of hindwings can be reconstructed based on phylogenetic analysis [9].

Although the method presented in this study can be applied to the detection of hindwing landmarks in other beetle groups, it should be noted that the existing dataset is not comprehensive and cannot fully represent the diversity of beetle hindwing morphology [32]. Further data collection and landmark annotation is still required to obtain a more comprehensive beetle hindwing training dataset for the proposed method, which would provide richer landmark data for the study of beetle wing morphology, function, evolution, and diversity.

In summary, this study has important implications for the field of coleopterology and other fields. We provided a new method for obtaining quantitative data on beetle hindwing morphology, and which can be further applied to the study of other insect wings. Further data collection and research are still required to improve the wing landmark detection accuracy within specific areas, extend the species application range of the proposed method, and understand the diversity and evolution of beetle wing morphology. The combination of HRNet and transfer learning provides a promising foundation for such future research.

## 5. Conclusions

In this study, the HRNet architecture fine-tuned on a specific domain through transfer learning demonstrated stronger leaf beetle hindwing landmark detection ability, with a lower dependence on the size of the training dataset compared to the current popular method. The proposed approach greatly improved the detection performance of convolutional neural networks in tasks with limited training data. When the model was trained on a small dataset of 100 hindwing images, the average detection error on 36 hindwing landmarks was less than 0.02, while the error only reached 0.045 when the training set was reduced to 3 hindwing samples. The proposed approach can significantly reduce the time spent by entomologists on landmark labeling, helping to accelerate research aimed at better understanding beetle evolution and gaining insights into key parameters for the optimization of biomimetic beetle-inspired micro aerial vehicles. This study provides a new method for obtaining quantitative data on beetle hindwing morphology, which can be further applied to the study of a wider range of insect wings. Further data collection and research should be carried out to improve the detection accuracy for specific landmarks on beetle wings, expand its application range to more insects, and better understand the diversity and evolution of insect wing morphology.

## Figures and Tables

**Table 1:** Basic information about the 36 landmarks of leaf beetle hindwings.

Landmark Index	Position Description
1	Proximal anterior point of humeral plate (HP)
2	The crossing point of BSc and Sc
3	The point of Sc getting to bifurcate into ScA and ScP
4	The crossing point of ScP and RA
5	The crossing point of ScA and RA
6	The crossing point of rp-m1 and RA
7	Proximal anterior point of radial cell
8	Distal anterior point of radial cell
9	Distal posterior point of radial cell
10	Anterior point of r4 (or the crossing point of r4 and radial cell)
11	Proximal posterior point of radial cell
12	Proximal point of r3
13	Apical hinge
14	The anterior point of triangular area of radial cell’s distal side
15	The posterior point of triangular area of radial cell’s distal side
16	The proximal point of triangular area of radial cell’s distal side
17	The distal point of RA_4
18	The distal point of RA_1
19	The distal point of RP_2
20	The point of MP_1+2_ getting to bifurcate
21	The posterior point of r4, or the crossing point of r4 and rp-mp2
22	The proximal point of RP
23	Anterior point of mp-cua
24	The crossing point of rm-mp1and MP
25	The posterior of medial spur
26	Posterior point of mp-cua
27	The point of AA getting to bifurcate
28	The point of AA_1+2_ getting to fuse with CuA_3+4_
29	The posterior or distal point of AA_3+4_
30	The proximal point of cv
31	The posterior or distal point of AA_1+2_+CuA_3+4_
32	Anterior point of CuA1+2+MP4
33	The distal point of cv
34	Posterior point of CuA_1+2_+MP_4
35	The base point of AP_3+4_
36	The posterior point of AP_3+4_

**Table 2:** Three strategies to fine-tune the parameters of the HRNet model.

Strategies	Stage1	Stage2	Stage3	Stage4	Hh (Heatmap Head)
TH-HRNet	✕	✕	✕	✕	✓
	✕	✕	✕	✓	✓
TS-HRNet	✕	✕	✓	✓	✓
	✕	✓	✓	✓	✓
TA-HRNet	✓	✓	✓	✓	✓

Notes: ✕ indicates the parameters of the stage were retained while ✓ indicates they were retrained.

## Data Availability

The data and code applied in this research is accessible through the following link: https://gitlab.com/mgcyung/hrnet-wing.git (accessed on 13 July 2023).

## Supplementary Materials

The following are available online at https://www.mdpi.com/article/10.3390/biology12071006/s1.
