# Supplementary material for: Detection of Hindwing Landmarks Using Transfer Learning and High-Resolution Networks
Source: Biology (Basel). 2023 Jul 14;12(7):1006. doi: 10.3390/biology12071006 (PMC10376506; doi:10.3390/biology12071006)
Supplement: Supplementary file 1 [file biology-12-01006-s001.zip › biology-2394554-supplementary.pdf]

Supplementary Materials:

Table S1. NME on 36 landmarks of leaf beetle hindwings using four training methods.

Training methods	TA-HRNet	**TS-HRNet**	TH-HRNet	HRNet
1	0.0248 ± 0.0007	0.0244 ± 0.0008	0.0261 ± 0.0004	0.0252 ± 0.0007
2	0.0175 ± 0.0004	0.0171 ± 0.0008	0.0173 ± 0.0005	0.0175 ± 0.0006
3	0.0179 ± 0.0006	0.0182 ± 0.0010	0.0226 ± 0.0007	0.0204 ± 0.0019
4	0.0234 ± 0.0007	0.0233 ± 0.0020	0.0295 ± 0.0008	0.0254 ± 0.0017
5	0.0185 ± 0.0010	0.0193 ± 0.0011	0.0273 ± 0.0016	0.0234 ± 0.0018
6	0.0120 ± 0.0006	0.0123 ± 0.0004	0.0153 ± 0.0004	0.0130 ± 0.0007
7	0.0147 ± 0.0005	0.0148 ± 0.0007	0.0207 ± 0.0008	0.0177 ± 0.0007
8	0.0150 ± 0.0006	0.0152 ± 0.0006	0.0239 ± 0.0007	0.0167 ± 0.0008
9	0.0168 ± 0.0009	0.0171 ± 0.0009	0.0226 ± 0.0006	0.0180 ± 0.0011
10	0.0186 ± 0.0006	0.0179 ± 0.0008	0.0237 ± 0.0007	0.0204 ± 0.0008
11	0.0163 ± 0.0007	0.0165 ± 0.0007	0.0246 ± 0.0011	0.0226 ± 0.0011
12	0.0150 ± 0.0008	0.0153 ± 0.0006	0.0187 ± 0.0007	0.0221 ± 0.0010
13	0.0135 ± 0.0006	0.0132 ± 0.0006	0.0193 ± 0.0011	0.0149 ± 0.0008
14	0.0135 ± 0.0004	0.0137 ± 0.0005	0.0155 ± 0.0003	0.0149 ± 0.0008
15	0.0149 ± 0.0006	0.0147 ± 0.0006	0.0189 ± 0.0007	0.0169 ± 0.0015
16	0.0130 ± 0.0006	0.0129 ± 0.0006	0.0204 ± 0.0024	0.0144 ± 0.0010
17	0.0283 ± 0.0018	0.0278 ± 0.0016	0.0419 ± 0.0028	0.0336 ± 0.0026
18	0.0246 ± 0.0007	0.0242 ± 0.0012	0.0357 ± 0.0012	0.0289 ± 0.0017
19	0.0168 ± 0.0014	0.0171 ± 0.0010	0.0260 ± 0.0008	0.0185 ± 0.0016
20	0.0117 ± 0.0002	0.0118 ± 0.0003	0.0160 ± 0.0004	0.0146 ± 0.0010
21	0.0189 ± 0.0008	0.0192 ± 0.0011	0.0255 ± 0.0014	0.0223 ± 0.0007
22	0.0248 ± 0.0033	0.0248 ± 0.0015	0.0401 ± 0.0009	0.0434 ± 0.0037
23	0.0121 ± 0.0003	0.0115 ± 0.0003	0.0142 ± 0.0007	0.0122 ± 0.0004
24	0.0124 ± 0.0005	0.0125 ± 0.0007	0.0166 ± 0.0005	0.0136 ± 0.0005
25	0.0168 ± 0.0009	0.0170 ± 0.0007	0.0294 ± 0.0021	0.0187 ± 0.0009
26	0.0129 ± 0.0008	0.0127 ± 0.0005	0.0151 ± 0.0008	0.0134 ± 0.0005
27	0.0181 ± 0.0015	0.0180 ± 0.0016	0.0260 ± 0.0061	0.0234 ± 0.0016
28	0.0199 ± 0.0021	0.0208 ± 0.0017	0.0285 ± 0.0054	0.0258 ± 0.0017
29	0.0225 ± 0.0009	0.0236 ± 0.0015	0.0385 ± 0.0056	0.0337 ± 0.0022
30	0.0327 ± 0.0036	0.0330 ± 0.0020	0.0462 ± 0.0056	0.0468 ± 0.0057
31	0.0190 ± 0.0008	0.0199 ± 0.0006	0.0340 ± 0.0042	0.0230 ± 0.0012
32	0.0304 ± 0.0042	0.0280 ± 0.0018	0.0433 ± 0.0036	0.0392 ± 0.0030
33	0.0466 ± 0.0026	0.0419 ± 0.0037	0.0633 ± 0.0038	0.0510 ± 0.0036
34	0.0199 ± 0.0016	0.0212 ± 0.0013	0.0467 ± 0.0036	0.0356 ± 0.0027
35	0.0169 ± 0.0004	0.0162 ± 0.0005	0.0202 ± 0.0004	0.0171 ± 0.0005
36	0.0275 ± 0.0009	0.0282 ± 0.0008	0.0310 ± 0.0015	0.0309 ± 0.0022
average	0.0194 ± 0.0072	0.0193 ± 0.0066	0.0274 ± 0.0112	0.0236 ± 0.0099

Table S2. NME on 36 landmarks of leaf beetle hindwings using TS-HRNet.

Retrained layers in TS-HRNet	Stage1-Stage4	**Stage2-Stage4**	Stage3-Stage4	Stage4
1	0.0248 ± 0.0007	0.0244 ± 0.0008	0.0244 ± 0.0008	0.0250 ± 0.0007
2	0.0175 ± 0.0004	0.0171 ± 0.0008	0.0179 ± 0.0006	0.0169 ± 0.0005
3	0.0179 ± 0.0006	0.0182 ± 0.0010	0.0188 ± 0.0005	0.0194 ± 0.0006
4	0.0234 ± 0.0007	0.0233 ± 0.0020	0.0237 ± 0.0012	0.0243 ± 0.0011
5	0.0185 ± 0.0010	0.0193 ± 0.0011	0.0200 ± 0.0006	0.0218 ± 0.0011
6	0.0120 ± 0.0006	0.0123 ± 0.0004	0.0124 ± 0.0005	0.0131 ± 0.0004
7	0.0147 ± 0.0005	0.0148 ± 0.0007	0.0147 ± 0.0006	0.0161 ± 0.0009
8	0.0150 ± 0.0006	0.0152 ± 0.0006	0.0153 ± 0.0005	0.0154 ± 0.0006
9	0.0168 ± 0.0009	0.0171 ± 0.0009	0.0168 ± 0.0007	0.0172 ± 0.0007
10	0.0186 ± 0.0006	0.0179 ± 0.0008	0.0182 ± 0.0008	0.0186 ± 0.0007
11	0.0163 ± 0.0007	0.0165 ± 0.0007	0.0167 ± 0.0007	0.0190 ± 0.0008
12	0.0150 ± 0.0008	0.0153 ± 0.0006	0.0153 ± 0.0004	0.0170 ± 0.0008
13	0.0135 ± 0.0006	0.0132 ± 0.0006	0.0132 ± 0.0004	0.0140 ± 0.0005
14	0.0135 ± 0.0004	0.0137 ± 0.0005	0.0139 ± 0.0004	0.0138 ± 0.0005
15	0.0149 ± 0.0006	0.0147 ± 0.0006	0.0144 ± 0.0006	0.0155 ± 0.0005
16	0.0130 ± 0.0006	0.0129 ± 0.0006	0.0130 ± 0.0006	0.0135 ± 0.0004
17	0.0283 ± 0.0018	0.0278 ± 0.0016	0.0280 ± 0.0011	0.0296 ± 0.0009
18	0.0246 ± 0.0007	0.0242 ± 0.0012	0.0244 ± 0.0007	0.0253 ± 0.0010
19	0.0168 ± 0.0014	0.0171 ± 0.0010	0.0179 ± 0.0009	0.0171 ± 0.0011
20	0.0117 ± 0.0002	0.0118 ± 0.0003	0.0120 ± 0.0005	0.0126 ± 0.0004
21	0.0189 ± 0.0008	0.0192 ± 0.0011	0.0190 ± 0.0009	0.0200 ± 0.0005
22	0.0248 ± 0.0033	0.0248 ± 0.0015	0.0254 ± 0.0034	0.0295 ± 0.0013
23	0.0121 ± 0.0003	0.0115 ± 0.0003	0.0117 ± 0.0005	0.0117 ± 0.0003
24	0.0124 ± 0.0005	0.0125 ± 0.0007	0.0126 ± 0.0004	0.0133 ± 0.0007
25	0.0168 ± 0.0009	0.0170 ± 0.0007	0.0168 ± 0.0003	0.0174 ± 0.0005
26	0.0129 ± 0.0008	0.0127 ± 0.0005	0.0127 ± 0.0006	0.0129 ± 0.0004
27	0.0181 ± 0.0015	0.0180 ± 0.0016	0.0185 ± 0.0012	0.0191 ± 0.0016
28	0.0199 ± 0.0021	0.0208 ± 0.0017	0.0219 ± 0.0011	0.0206 ± 0.0009
29	0.0225 ± 0.0009	0.0236 ± 0.0015	0.0242 ± 0.0012	0.0269 ± 0.0021
30	0.0327 ± 0.0036	0.0330 ± 0.0020	0.0351 ± 0.0046	0.0402 ± 0.0031
31	0.0190 ± 0.0008	0.0199 ± 0.0006	0.0197 ± 0.0006	0.0203 ± 0.0006
32	0.0304 ± 0.0042	0.0280 ± 0.0018	0.0290 ± 0.0019	0.0279 ± 0.0028
33	0.0466 ± 0.0026	0.0419 ± 0.0037	0.0466 ± 0.0051	0.0466 ± 0.0041
34	0.0199 ± 0.0016	0.0212 ± 0.0013	0.0220 ± 0.0013	0.0240 ± 0.0014
35	0.0169 ± 0.0004	0.0162 ± 0.0005	0.0163 ± 0.0007	0.0167 ± 0.0004
36	0.0275 ± 0.0009	0.0282 ± 0.0008	0.0280 ± 0.0006	0.0293 ± 0.0014
average	0.0194 ± 0.0072	0.0193 ± 0.0066	0.0197 ± 0.0073	0.0206 ± 0.0077

Table S3. Influence of different numbers of training samples on the performance of TS-HRNet.

Numbers	1	3	5	10	50	100
1	0.0911 ± 0.0696	0.0326 ± 0.0074	0.0307 ± 0.0056	0.0270 ± 0.0013	0.0248 ± 0.0007	0.0244 ± 0.0008
2	0.0711 ± 0.0740	0.0199 ± 0.0037	0.0192 ± 0.0022	0.0184 ± 0.0017	0.0175 ± 0.0011	0.0171 ± 0.0008
3	0.0915 ± 0.0527	0.0278 ± 0.0058	0.0266 ± 0.0074	0.0232 ± 0.0024	0.0194 ± 0.0010	0.0182 ± 0.0010
4	0.1357 ± 0.0723	0.0441 ± 0.0094	0.0393 ± 0.0052	0.0330 ± 0.0049	0.0256 ± 0.0018	0.0233 ± 0.0020
5	0.1820 ± 0.0941	0.0383 ± 0.0104	0.0330 ± 0.0065	0.0255 ± 0.0045	0.0213 ± 0.0011	0.0193 ± 0.0011
6	0.0603 ± 0.0337	0.0213 ± 0.0044	0.0200 ± 0.0030	0.0159 ± 0.0016	0.0138 ± 0.0011	0.0123 ± 0.0004
7	0.1198 ± 0.0708	0.0459 ± 0.0124	0.0314 ± 0.0054	0.0188 ± 0.0031	0.0150 ± 0.0006	0.0148 ± 0.0007
8	0.1232 ± 0.0723	0.0354 ± 0.0093	0.0285 ± 0.0052	0.0192 ± 0.0018	0.0162 ± 0.0007	0.0152 ± 0.0006
9	0.1032 ± 0.0558	0.0383 ± 0.0124	0.0284 ± 0.0060	0.0215 ± 0.0037	0.0173 ± 0.0007	0.0171 ± 0.0009
10	0.1335 ± 0.0474	0.0404 ± 0.0086	0.0326 ± 0.0059	0.0249 ± 0.0035	0.0208 ± 0.0012	0.0179 ± 0.0008
11	0.1377 ± 0.0540	0.0410 ± 0.0095	0.0297 ± 0.0059	0.0236 ± 0.0027	0.0172 ± 0.0015	0.0165 ± 0.0007
12	0.1107 ± 0.0546	0.0283 ± 0.0080	0.0227 ± 0.0046	0.0211 ± 0.0032	0.0157 ± 0.0006	0.0153 ± 0.0006
13	0.1528 ± 0.0758	0.0277 ± 0.0083	0.0264 ± 0.0101	0.0168 ± 0.0015	0.0136 ± 0.0006	0.0132 ± 0.0006
14	0.1266 ± 0.0871	0.0326 ± 0.0090	0.0215 ± 0.0061	0.0148 ± 0.0008	0.0142 ± 0.0005	0.0137 ± 0.0005
15	0.1171 ± 0.0501	0.0296 ± 0.0066	0.0292 ± 0.0115	0.0175 ± 0.0013	0.0150 ± 0.0005	0.0147 ± 0.0006
16	0.0940 ± 0.0303	0.0409 ± 0.0074	0.0257 ± 0.0083	0.0182 ± 0.0049	0.0136 ± 0.0006	0.0129 ± 0.0006
17	0.1201 ± 0.0408	0.0539 ± 0.0100	0.0483 ± 0.0123	0.0402 ± 0.0065	0.0303 ± 0.0012	0.0278 ± 0.0016
18	0.0683 ± 0.0224	0.0461 ± 0.0107	0.0384 ± 0.0045	0.0327 ± 0.0035	0.0263 ± 0.0019	0.0242 ± 0.0012
19	0.0955 ± 0.0561	0.0393 ± 0.0072	0.0338 ± 0.0108	0.0245 ± 0.0037	0.0190 ± 0.0011	0.0171 ± 0.0010
20	0.0641 ± 0.0332	0.0232 ± 0.0058	0.0183 ± 0.0039	0.0140 ± 0.0014	0.0125 ± 0.0006	0.0118 ± 0.0003
21	0.1043 ± 0.0384	0.0403 ± 0.0083	0.0352 ± 0.0065	0.0253 ± 0.0022	0.0202 ± 0.0011	0.0192 ± 0.0011
22	0.1029 ± 0.0321	0.0493 ± 0.0077	0.0505 ± 0.0111	0.0422 ± 0.0025	0.0326 ± 0.0033	0.0248 ± 0.0015
23	0.0406 ± 0.0207	0.0196 ± 0.0035	0.0174 ± 0.0020	0.0139 ± 0.0009	0.0123 ± 0.0006	0.0115 ± 0.0003
24	0.0725 ± 0.0517	0.0177 ± 0.0029	0.0175 ± 0.0030	0.0149 ± 0.0008	0.0133 ± 0.0008	0.0125 ± 0.0007
25	0.2307 ± 0.1023	0.0581 ± 0.0172	0.0323 ± 0.0080	0.0198 ± 0.0023	0.0172 ± 0.0011	0.0170 ± 0.0007
26	0.0441 ± 0.0151	0.0187 ± 0.0033	0.0164 ± 0.0013	0.0150 ± 0.0011	0.0134 ± 0.0005	0.0127 ± 0.0005
27	0.1591 ± 0.0658	0.0756 ± 0.0306	0.0407 ± 0.0133	0.0299 ± 0.0062	0.0191 ± 0.0016	0.0180 ± 0.0016
28	0.1440 ± 0.0540	0.0757 ± 0.0332	0.0443 ± 0.0157	0.0310 ± 0.0049	0.0221 ± 0.0016	0.0208 ± 0.0017
29	0.1938 ± 0.0622	0.0747 ± 0.0205	0.0583 ± 0.0094	0.0473 ± 0.0057	0.0272 ± 0.0047	0.0236 ± 0.0015
30	0.2074 ± 0.0627	0.0875 ± 0.0259	0.0873 ± 0.0317	0.0601 ± 0.0099	0.0385 ± 0.0048	0.0330 ± 0.0020
31	0.1653 ± 0.0477	0.0605 ± 0.0263	0.0448 ± 0.0082	0.0337 ± 0.0082	0.0204 ± 0.0009	0.0199 ± 0.0006
32	0.1447 ± 0.0541	0.0652 ± 0.0133	0.0685 ± 0.0211	0.0455 ± 0.0093	0.0349 ± 0.0048	0.0280 ± 0.0018
33	0.1896 ± 0.0712	0.1078 ± 0.0304	0.1074 ± 0.0350	0.0886 ± 0.0341	0.0560 ± 0.0075	0.0419 ± 0.0037
34	0.2050 ± 0.0563	0.0918 ± 0.0430	0.0678 ± 0.0297	0.0448 ± 0.0106	0.0239 ± 0.0014	0.0212 ± 0.0013
35	0.0572 ± 0.0298	0.0224 ± 0.0023	0.0220 ± 0.0021	0.0202 ± 0.0012	0.0165 ± 0.0011	0.0162 ± 0.0005
36	0.1663 ± 0.1133	0.0590 ± 0.0305	0.0372 ± 0.0027	0.0329 ± 0.0021	0.0290 ± 0.0012	0.0282 ± 0.0008
average	0.1229 ± 0.0750	0.0453 ± 0.0271	0.0370 ± 0.0228	0.0282 ± 0.0165	0.0213 ± 0.0091	0.0193 ± 0.0066

Table S4. Influence of different numbers of training samples on the performance of TS-ResNet.

Numbers	1	3	5	10	50	100
1	0.1052 ± 0.0532	0.0576 ± 0.0130	0.0515 ± 0.0136	0.0418 ± 0.0066	0.0297 ± 0.0014	0.0266 ± 0.0010
2	0.1126 ± 0.0552	0.0481 ± 0.0176	0.0391 ± 0.0074	0.0278 ± 0.0031	0.0205 ± 0.0008	0.0189 ± 0.0004
3	0.1049 ± 0.0385	0.0638 ± 0.0247	0.0476 ± 0.0193	0.0379 ± 0.0060	0.0241 ± 0.0013	0.0220 ± 0.0009
4	0.1038 ± 0.0278	0.0687 ± 0.0120	0.0732 ± 0.0332	0.0488 ± 0.0037	0.0306 ± 0.0011	0.0279 ± 0.0016
5	0.1110 ± 0.0283	0.0788 ± 0.0118	0.0883 ± 0.0226	0.0610 ± 0.0139	0.0327 ± 0.0025	0.0284 ± 0.0011
6	0.1230 ± 0.0563	0.0482 ± 0.0162	0.0337 ± 0.0043	0.0277 ± 0.0041	0.0161 ± 0.0011	0.0146 ± 0.0005
7	0.1504 ± 0.0436	0.0757 ± 0.0144	0.0787 ± 0.0219	0.0389 ± 0.0042	0.0230 ± 0.0014	0.0198 ± 0.0006
8	0.1572 ± 0.0613	0.0702 ± 0.0092	0.0744 ± 0.0169	0.0402 ± 0.0034	0.0210 ± 0.0015	0.0181 ± 0.0005
9	0.1467 ± 0.0574	0.0721 ± 0.0087	0.0742 ± 0.0219	0.0389 ± 0.0040	0.0211 ± 0.0010	0.0190 ± 0.0008
10	0.1312 ± 0.0474	0.0760 ± 0.0135	0.0696 ± 0.0135	0.0409 ± 0.0038	0.0296 ± 0.0016	0.0251 ± 0.0012
11	0.1371 ± 0.0455	0.0775 ± 0.0178	0.0763 ± 0.0282	0.0370 ± 0.0031	0.0284 ± 0.0019	0.0250 ± 0.0013
12	0.1241 ± 0.0465	0.0704 ± 0.0056	0.0757 ± 0.0268	0.0400 ± 0.0037	0.0273 ± 0.0019	0.0239 ± 0.0009
13	0.1885 ± 0.0892	0.0836 ± 0.0206	0.0851 ± 0.0199	0.0408 ± 0.0048	0.0199 ± 0.0013	0.0167 ± 0.0008
14	0.1436 ± 0.0544	0.0694 ± 0.0082	0.0687 ± 0.0151	0.0377 ± 0.0030	0.0198 ± 0.0015	0.0170 ± 0.0007
15	0.1380 ± 0.0528	0.0824 ± 0.0196	0.1076 ± 0.0347	0.0451 ± 0.0065	0.0216 ± 0.0012	0.0179 ± 0.0005
16	0.1353 ± 0.0450	0.0668 ± 0.0043	0.0693 ± 0.0116	0.0380 ± 0.0034	0.0189 ± 0.0015	0.0163 ± 0.0005
17	0.1553 ± 0.0779	0.1322 ± 0.1556	0.1000 ± 0.0487	0.0543 ± 0.0163	0.0355 ± 0.0030	0.0334 ± 0.0016
18	0.1482 ± 0.0747	0.1001 ± 0.0528	0.1251 ± 0.1276	0.0543 ± 0.0115	0.0358 ± 0.0026	0.0320 ± 0.0021
19	0.1966 ± 0.0979	0.2171 ± 0.1560	0.2218 ± 0.1285	0.1047 ± 0.0501	0.0313 ± 0.0027	0.0258 ± 0.0035
20	0.1464 ± 0.0449	0.0948 ± 0.0260	0.1056 ± 0.0275	0.0464 ± 0.0060	0.0244 ± 0.0021	0.0195 ± 0.0010
21	0.1141 ± 0.0260	0.0767 ± 0.0137	0.0779 ± 0.0153	0.0428 ± 0.0047	0.0279 ± 0.0013	0.0245 ± 0.0009
22	0.1179 ± 0.0306	0.0811 ± 0.0064	0.0859 ± 0.0174	0.0609 ± 0.0063	0.0495 ± 0.0018	0.0444 ± 0.0019
23	0.1203 ± 0.0597	0.0452 ± 0.0164	0.0332 ± 0.0049	0.0247 ± 0.0030	0.0159 ± 0.0009	0.0136 ± 0.0004
24	0.1239 ± 0.0537	0.0444 ± 0.0156	0.0312 ± 0.0020	0.0255 ± 0.0029	0.0160 ± 0.0009	0.0147 ± 0.0006
25	0.1906 ± 0.0533	0.1865 ± 0.0856	0.1912 ± 0.0964	0.0681 ± 0.0134	0.0298 ± 0.0023	0.0243 ± 0.0014
26	0.1079 ± 0.0469	0.0437 ± 0.0151	0.0324 ± 0.0034	0.0245 ± 0.0021	0.0160 ± 0.0008	0.0148 ± 0.0004
27	0.0966 ± 0.0289	0.0649 ± 0.0156	0.0602 ± 0.0251	0.0405 ± 0.0037	0.0300 ± 0.0016	0.0264 ± 0.0008
28	0.0994 ± 0.0272	0.0608 ± 0.0127	0.0541 ± 0.0158	0.0411 ± 0.0032	0.0305 ± 0.0015	0.0282 ± 0.0010
29	0.1533 ± 0.0583	0.0823 ± 0.0119	0.0904 ± 0.0287	0.0597 ± 0.0073	0.0457 ± 0.0036	0.0380 ± 0.0022
30	0.1549 ± 0.0494	0.0928 ± 0.0130	0.0833 ± 0.0116	0.0775 ± 0.0126	0.0557 ± 0.0049	0.0498 ± 0.0017
31	0.1548 ± 0.0508	0.0836 ± 0.0199	0.0802 ± 0.0286	0.0512 ± 0.0078	0.0344 ± 0.0034	0.0297 ± 0.0009
32	0.1397 ± 0.0449	0.0850 ± 0.0101	0.0799 ± 0.0130	0.0661 ± 0.0184	0.0477 ± 0.0020	0.0433 ± 0.0024
33	0.1766 ± 0.0537	0.1079 ± 0.0206	0.1083 ± 0.0221	0.0946 ± 0.0203	0.0648 ± 0.0057	0.0551 ± 0.0033
34	0.1729 ± 0.0357	0.1212 ± 0.0330	0.1254 ± 0.0298	0.0899 ± 0.0211	0.0475 ± 0.0029	0.0409 ± 0.0021
35	0.1045 ± 0.0435	0.0494 ± 0.0158	0.0372 ± 0.0073	0.0270 ± 0.0022	0.0191 ± 0.0007	0.0178 ± 0.0004
36	0.0982 ± 0.0472	0.0829 ± 0.0391	0.0629 ± 0.0185	0.0468 ± 0.0041	0.0404 ± 0.0030	0.0368 ± 0.0023
average	0.1357 ± 0.0570	0.0823 ± 0.0544	0.0805 ± 0.0547	0.0484 ± 0.0223	0.0301 ± 0.0120	0.0264 ± 0.0104
